# Encrustations on ureteral stents from patients without urinary tract infection reveal distinct urotypes and a low bacterial load

**DOI:** 10.1186/s40168-019-0674-x

**Published:** 2019-04-13

**Authors:** Matthias T. Buhmann, Dominik Abt, Oliver Nolte, Thomas R. Neu, Sebastian Strempel, Werner C. Albrich, Patrick Betschart, Valentin Zumstein, Antonia Neels, Katharina Maniura-Weber, Qun Ren

**Affiliations:** 1 Laboratory for Biointerfaces, Empa, Swiss Federal Laboratories for Materials Science and Technology, Lerchenfeldstrasse 5, 9014 St. Gallen, Switzerland; 2 Department of Urology, Kantonsspital St. Gallen, Rorschacherstrasse 95, 9007 St. Gallen, Switzerland; 3 Zentrum für Labormedizin, Frohbergstrasse 3, 9001 St. Gallen, Switzerland; 4 Division of Infectious Diseases/Hospital Epidemiology, Kantonsspital St. Gallen, Rorschacherstrasse 95, 9007 St. Gallen, Switzerland; 5 Microsynth AG, Schützenstrasse 15, 9436 Balgach, Switzerland; 6 Microbiology of Interfaces, Department River Ecology, Helmholtz Centre for Environmental Research – UFZ, Brueckstrasse 3A, 39114 Magdeburg, Germany; 7 Center for X-ray Analytics, Empa, Überlandstrasse 129, 8600 Dübendorf, Switzerland

**Keywords:** Urinary tract microbiota, Ureteral stent, Encrustation, Biofilm, Next-generation sequencing, Cultivation-independent methods, qPCR, Microbiome

## Abstract

**Background:**

Current knowledge of the urinary tract microbiome is limited to urine analysis and analysis of biofilms formed on Foley catheters. Bacterial biofilms on ureteral stents have rarely been investigated, and no cultivation-independent data are available on the microbiome of the encrustations on the stents.

**Results:**

The typical encrustations of organic and inorganic urine-derived material, including microbial biofilms formed during 3–6 weeks on ureteral stents in patients treated for kidney and ureteral stones, and without reported urinary tract infection at the time of stent insertion, were analysed. Next-generation sequencing of the 16S rRNA gene V3–V4 region revealed presence of different urotypes, distinct bacterial communities. Analysis of bacterial load was performed by combining quantification of 16S rRNA gene copy numbers by qPCR with microscopy and cultivation-dependent analysis methods, which revealed that ureteral stent biofilms mostly contain low numbers of bacteria. Fluorescence microscopy indicates the presence of extracellular DNA. Bacteria identified in biofilms by microscopy had mostly morphogenic similarities to gram-positive bacteria, in few cases to *Lactobacillus* and *Corynebacterium*, while sequencing showed many additional bacterial genera. Weddellite crystals were absent in biofilms of patients with *Enterobacterales* and *Corynebacterium-*dominated microbiomes.

**Conclusions:**

This study provides novel insights into the bacterial burden in ureteral stent encrustations and the urinary tract microbiome. Short-term (3–6 weeks) ureteral stenting is associated with a low load of viable and visible bacteria in ureteral stent encrustations, which may be different from long-term stenting. Patients could be classified according to different urotypes, some of which were dominated by potentially pathogenic species*.* Facultative pathogens however appear to be a common feature in patients without clinically manifested urinary tract infection.

**Trial registration:**

ClinicalTrials.gov, NCT02845726. Registered on 30 June 2016—retrospectively registered.

**Electronic supplementary material:**

The online version of this article (10.1186/s40168-019-0674-x) contains supplementary material, which is available to authorized users.

## Background

Urine is not sterile and the urinary tract harbours its distinct microbiota which is different from the genital microbiota [1, 2]. The overall number of bacteria present in urine of healthy patients is regarded as being rather low due to the presence of antimicrobial defence mechanisms and unfavourable growth conditions [3, 4]. The risk for urinary tract infections (UTIs), which may lead to pyelonephritis and urosepsis [5], is potentiated in the presence of medical devices that provide a surface for bacterial biofilm formation [6]. The biofilm lifestyle may turn normally harmless, commensal bacteria into pathogens [7] and is associated with increased antimicrobial resistance [8].

Ureteral stenting is a common surgical procedure to treat ureteral obstruction, which is often caused by urinary stones or malignancy. Short-term stenting for stone removal (< 6 weeks) represents an immense economic burden [9]. When in contact with urine, ureteral stents become frequently covered by calcium phosphate and calcium oxalate crystal-containing encrustations which can contribute to damage to the uroepithelium and pain, and have been proposed to promote infections [10]. Certain urinary tract pathogens such as *Proteus mirabilis* and *Pseudomonas aeruginosa* are known to promote crystal growth [11].

The impact of biofilms and encrustations of ureteral stents on stent-associated symptoms has been discussed controversially [12, 13]. To date, little is known about the composition and abundance of the bacterial communities present on ureteral stents and their role in health and disease. Most knowledge of the urinary tract microbiome derives from urine samples and biofilms on Foley catheters, based on either cultivation-dependent [14–17] or cultivation-independent [1, 2, 18–23] microbiome profiling methods. No sequencing-based microbiome profiling of ureteral stent encrustations has been reported yet. Common urinary tract pathogens such as *Escherichia coli* or *Enterococcus* have been identified in ureteral stent encrustations [24], while nothing is known about the contribution of commensal bacteria, which require enhanced cultivation conditions and cannot be cultivated using standard cultivation protocols [14, 25, 26].

Due to the low number of bacteria in urine and the sensitivity of PCR-based methods including sequencing, the risk of contaminating bacteria masking sample microbiota is high [27, 28]. It has been recognized that the comparability of microbiome studies is severely influenced by the urine sampling technique, since voided urine specimen is prone to get contaminated by urethral and genital microbiota [16]. As such, attempts to avoid contamination with urethral microbiota have been undertaken by direct sampling of urine from the bladder via transurethral catheterization or suprapubic aspiration [1, 16].

Since bacterial biofilm communities differentiate and adjust to the surface-associated life [29], it remains questionable if existing information about the urinary microbiome may be transferred to the encrustations found on urinary biomaterials. Microbiomes are known to undergo a shift in community composition in situations of disease or antibiotic treatment, but it is not known if and which bacterial profiles correspond to health and whether the presence of facultative pathogenic bacteria in patients without UTI symptoms has clinical implications [15, 30]. Similarly, it is not known if certain commensal microbiota contributes to the formation of biofilms on urinary tract medical devices.

The aim of this study was to improve our understanding of the presence and abundance of bacteria in encrustations on ureteral stents and to identify possible correlations between microbiome profiles and sample or patient characteristics in a cohort of patients without urinary tract infection. We addressed these issues by performing a 16S rRNA gene sequencing-based microbial survey on clinical ureteral stent samples, and in parallel, characterizing the encrustations with complementary techniques such as cultivation, scanning electron microscopy (SEM), assessment of dominant crystalline phases via X-ray diffraction (XRD) analysis, urine analysis and documentation of patient characteristics. To the best of our knowledge, this is the first cultivation-independent study on this particular type of ureteral stent encrustations.

## Results

### Study design and patient cohort

This study investigated encrustations and biofilms formed on the surface of 89 ureteral stents from stone-forming patients of an average age of 54 ± 15 years (range 16–85 years), including 68 male and 21 female patients. Patient characteristics are summarized in Table 1. None of the patients had a urinary tract infection (UTI) or taken antibiotics 1 month before insertion of the stent. During a time span of 3–6 weeks after insertion, four patients had taken antibiotics. Consistent with clinical guidelines, a single-shot antibiotic (mostly cotrimoxazole; ciprofloxacin or ceftriaxone in exceptional cases) was administered to all patients 1 h before removal of the stent. The stents were removed endoscopically, followed by sampling for imaging and extraction of the encrustations, biomass quantification, cultivation of bacteria, qPCR-based assessment of the bacterial load and microbiome profiling based on sequencing of the V3–V4 region of the 16S rRNA gene. Using XRD, encrustations were analysed for dominant crystalline phases. Moreover, routine blood and urine analyses were performed before stent insertion and removal, including cultivation of midstream voided urine for man and urine collected via intermittent bladder catheterization for women as well as counting of blood and urine leukocytes and erythrocytes. Detailed patient and sample characteristics and relevant clinical data can be found in Additional file 1.

**Table 1:** Demographic and clinical data of the study participants (*N* = 89)

Average age	53.9 ± 14.8 (range 16–85)
Gender	68 male, 21 female
BMI (kg m^-2^)	26.8 ± 4.3
Diabetes	22/89
Indwelling time	4.1 ± 0.8 (range 3–6) weeks
Microbiome profiles generated	85 (89 samples sequenced, 4 samples below threshold)
Antibiotic intake during stent indwelling time	Patient samples ST09, ST24, ST76 and ST93

*BMI* body mass index

### Microscopical analysis of encrustations

**Fig. 1:**
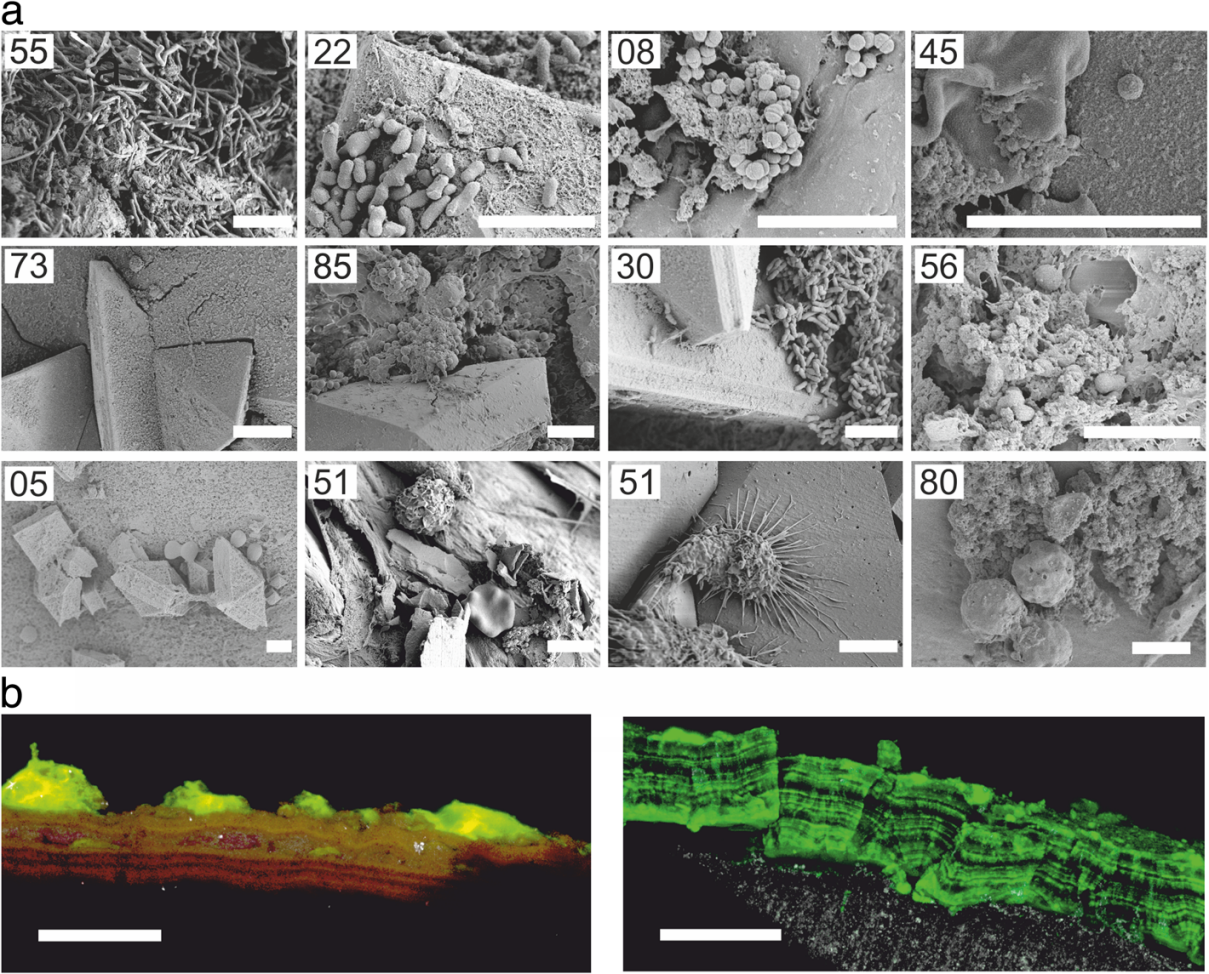
**a** Representative scanning electron micrographs of ureteral stent encrustations and biofilms. Numbers refer to sample IDs. Based on NGS and cultivation, bacteria visible likely include *L. jensenii* (ST55), *G. vaginalis* (ST22), *S. anginosus* or *A. tetradius* (ST08), *Staphylococcus* (ST45), *S. epidermidis* (ST85) and *Corynebacterium* (ST30). Further samples included structures with size and morphology of fungal cells or blood cells (ST05, ST51 and ST80). Scale bars = 5 μm. **b** Confocal laser scanning micrographs of SYBR Green-stained ureteral stent encrustations, shown as maximum intensity projection. Fluorescence was associated with a layered structure. Colour allocation—grey, reflection; green, SYBR Green. Scale bars = 50 μm

**Fig. 2:**
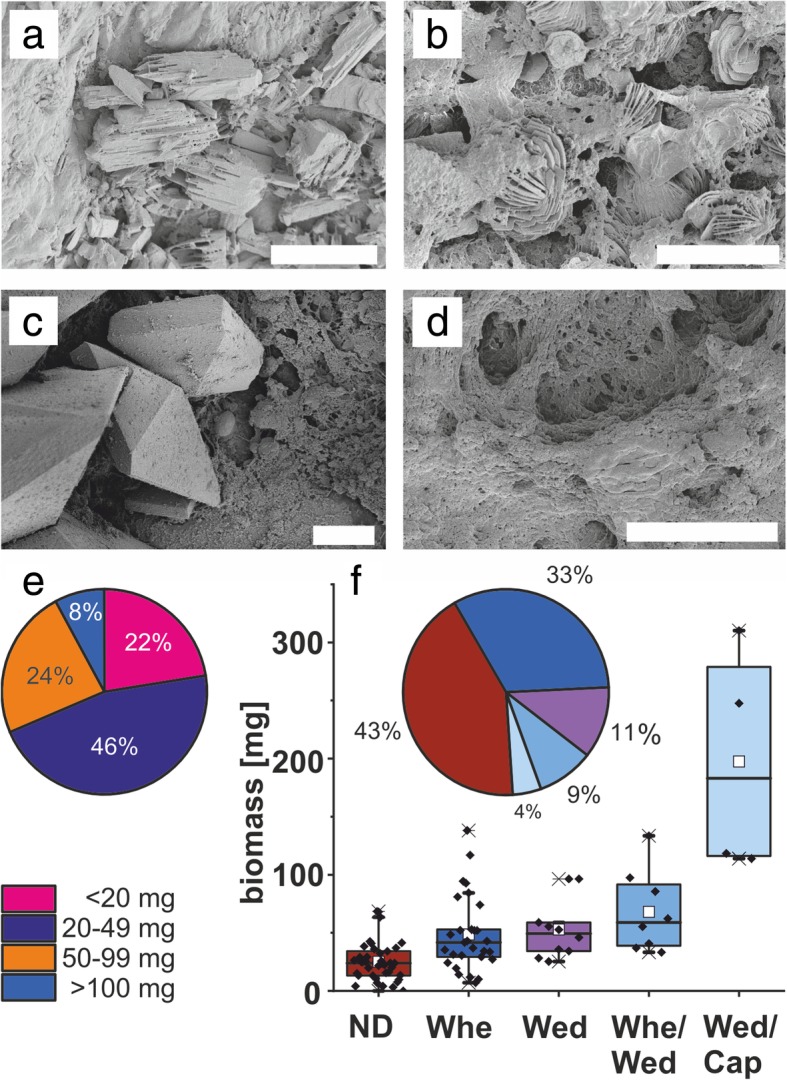
**a**–**d** Representative scanning electron micrographs of dominant crystalline phases in ureteral stent encrustations. Scale bars = 10 μm. **a** Dicalcium phosphate dihydrate (ST06). **b** Whewellite (ST16). **c** Weddellite (ST31). **d** Absence of crystals (no XRD signal), organic deposits (ST04). **e** Distribution of encrustation biomass. **f** Distribution of encrustation biomass among crystalline phases. *ND* absence of crystals, *Whe* whewellite, *Wed* weddellite, *Cap* dicalcium phosphate dihydrate

Small sections of both ureteral stent ends were imaged by SEM, while the rest of the encrustations were extracted from the stent surface. Imaging revealed that only a few samples exhibited bacteria-like structures (i.e. 14 out of 89) (e.g. samples ST55, ST22 and ST08 in Fig. 1a). The bulk of the encrustations appeared to consist of crystals or fibrous organic deposits (Fig. 2a–d). Several samples contained structures in the shape and size of blood cells, such as erythrocytes, leukocytes or macrophages (e.g. samples ST51 and ST80 in Fig. 1a). Clear evidence for fungal cells by both cultivation and microscopy has only been found for one sample (i.e. *Candida glabrata*, ST02; Additional file 2: Figure S1). Staining of the encrustations with fluorescent dyes and analysis via confocal laser scanning microscopy did not allow for visualization of bacteria in deeper layers of the encrustations due to intense autofluorescence of crystals (*not shown*). However, staining with SYBR Green I revealed staining of the encrustations, as well as fluorescent staining of discrete layers, indicative for the presence of extracellular DNA (eDNA) (Fig. 1b).

SEM imaging indicated thick crystal-containing layers in more than 50% of the samples, frequently in tetragonal dipyramidal shape indicative for weddellite (calcium oxalate dihydrate, CaC_2_O_4_·2H_2_O), or in prismatic shape indicative for whewellite (calcium oxalate monohydrate, CaC_2_O_4_·H_2_O) (Fig. 2b, c). In a low number of samples, however, crystalline material seemed to be absent according to SEM and XRD analyses, despite of high biomass, as determined by weighing the abraded encrustations (Fig. 2d, f).

### Quantification of bacterial load via 16S qPCR

**Fig. 3:**
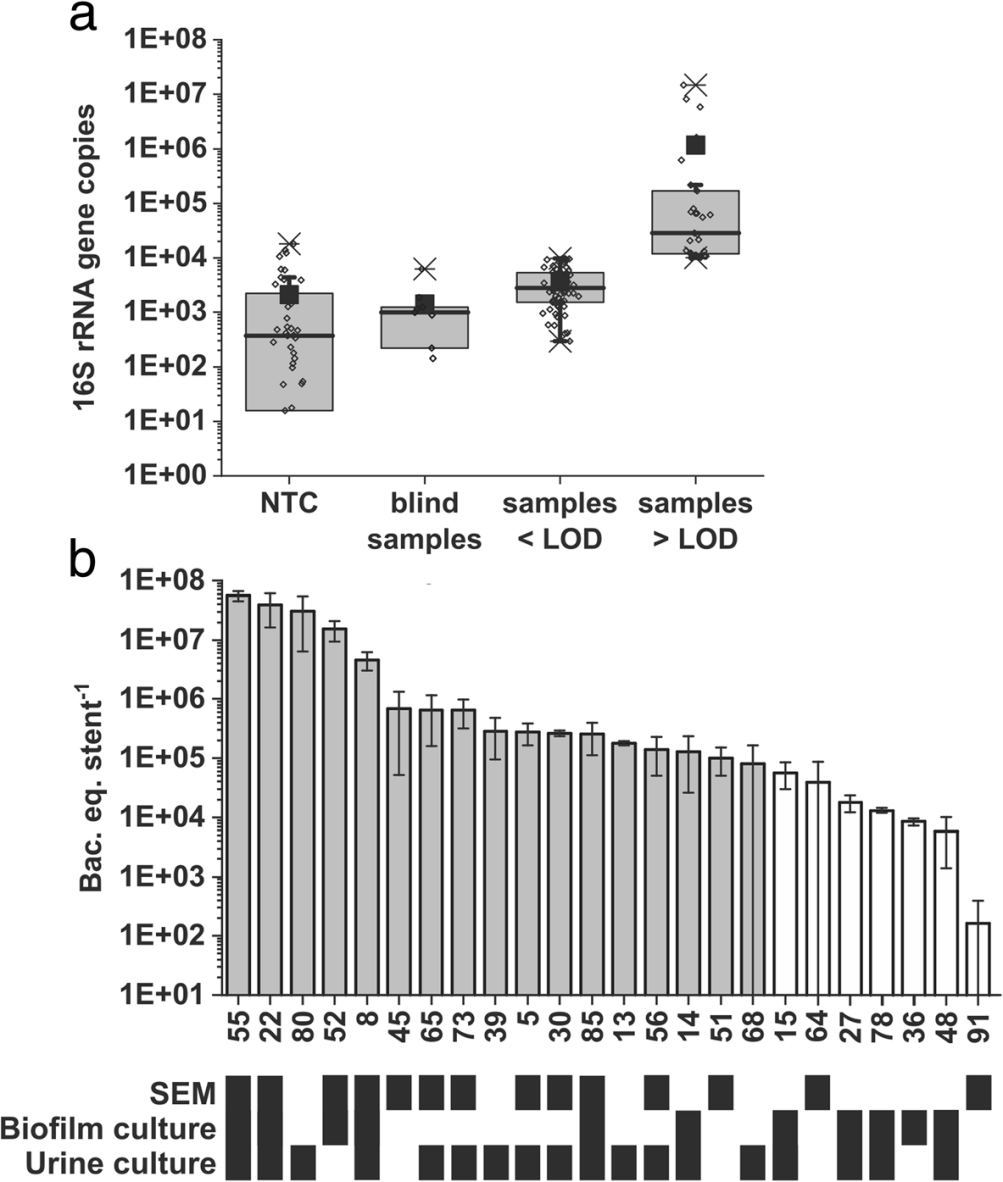
Assessment of bacterial load by 16S qPCR. **a** Detection of bacterial DNA in water controls (NTC), blind samples (unused ureteral stents) and samples below and above the limit of detection (LOD). Each data point (open diamonds) represents a PCR reaction. **b** Samples (IDs indicated on *y*-axis) with evidence for biofilm bacteria. Grey bars indicate samples above the LOD, white bars those below. Error bars represent the standard deviation between three technical qPCR replicates. The table below illustrates indications for the presence of bacteria (black boxes) according to analyses by SEM, and bacterial cultivation from encrustation or urine samples

To further assess the bacterial load quantitatively, the content of bacterial 16S rRNA genes was determined by universal 16S qPCR [31]. The qPCR assay allowed for a linear calibration curve over 7 orders of magnitude (Pearson’s *r* = 0.99, *R*-square 0.98, Additional file 2, Figure S2a,) and had a limit of detection (LOD) of 1.4 × 10^4^ 16S rRNA gene copies. Quantification of bacterial 16S rRNA genes confirmed that most samples contained very low amounts of bacterial DNA, close to the LOD of the assay. Less than a fifth of the samples (i.e. 17 out of 88) exceeded the LOD. All stent samples contained significantly higher amounts of bacterial DNA than water controls (Mann-Whitney *p* < 0.0001) or blind controls (Mann-Whitney *p* = 0.0020) (Fig. 3a).

In this work, encrustation is referred to the mixture of substances covering the ureteral stents and includes both microbial biofilms, as well as organic and inorganic deposits of biological origin. Biofilm is referred to aggregates of microbial cells embedded in a matrix of self-produced extracellular polymeric substances. For discrimination between biofilm bacteria and the signal derived from eDNA, bacterial load assessed by qPCR was compared with bacteria visible by SEM imaging, cultivation of resuspended encrustations and urine culture (Fig. 3b). Of all stent samples with a bacterial load above the LOD of the qPCR assay, 70.6% (12 out of 17) showed biofilm bacteria visible by SEM. Only one sample, ST91, exhibited a patch of visible bacteria even though bacterial DNA concentrations remained far below the LOD of the qPCR assay (Additional file 2: Figure S1).

### Compositional analysis of ureteral stent encrustations via next-generation sequencing

In addition to the quantitative assessment of the bacteria, we performed next-generation sequencing (NGS) for qualitative information on bacteria or bacterial DNA present in the encrustations. Sequencing of the V3–V4 region of the 16S rRNA gene yielded a total of 7,659,426 paired-end reads and a total of 3,828,181,036 pass filter bases. If possible, each paired-end read was assembled to one continuous sequence to represent the sequenced molecule, yielding 7,324,962 merged reads. Eighty-five out of 89 samples produced more merged reads than the cutoff of 1000 (ranging from 1382 to 380,850, median 38,111). Ureteral stent microbiome profiles were generated from a mean of 84,671 ± 119,303 reads per sample with assigned taxonomy and 16S rRNA gene copy number corrected abundance (hence the difference and inflation in certain samples, ranging from 256 to 618,050, median at 37,060). Across the 85 samples, 7,197,040 merged and 16S rRNA gene copy number-corrected reads were assigned to 164 operational taxonomic units (OTUs). On average 43,884 ± 95,992 reads were assigned to each OTU (ranging from 824 to 684,056; median, 6736), resulting in 164 unique OTUs. Of the 164 OTUs, 13 OTUs were commonly found in more than 50% of the samples; however, no single OTU was found to be commonly present in more than 90% of the samples (Additional file 2: Table S3). Each sample contained a mean of 31.28 ± 14.57 OTUs (ranging from 7 to 67, median at 30). Most of the sequences (i.e. 81%) could be assigned to the species level, 14% to the genus level, and 5% to the family level or higher taxa. A species was regarded as identified if having a confidence score of at least 0.7. Three no template controls and three blind samples were sequenced but excluded from the analyses due to too few sequencing reads and not passing quality control. Calculation of rarefaction plots of Chao1-estimated richness of species indicates that the number of sequenced reads per sample sufficiently represented the ureteral stent microbiota, since for most samples, the curves reached a plateau according to the rarefaction analysis (Additional file 2: Figure S4).

### Clustering reveals distinct microbiome profiles

**Fig. 4:**
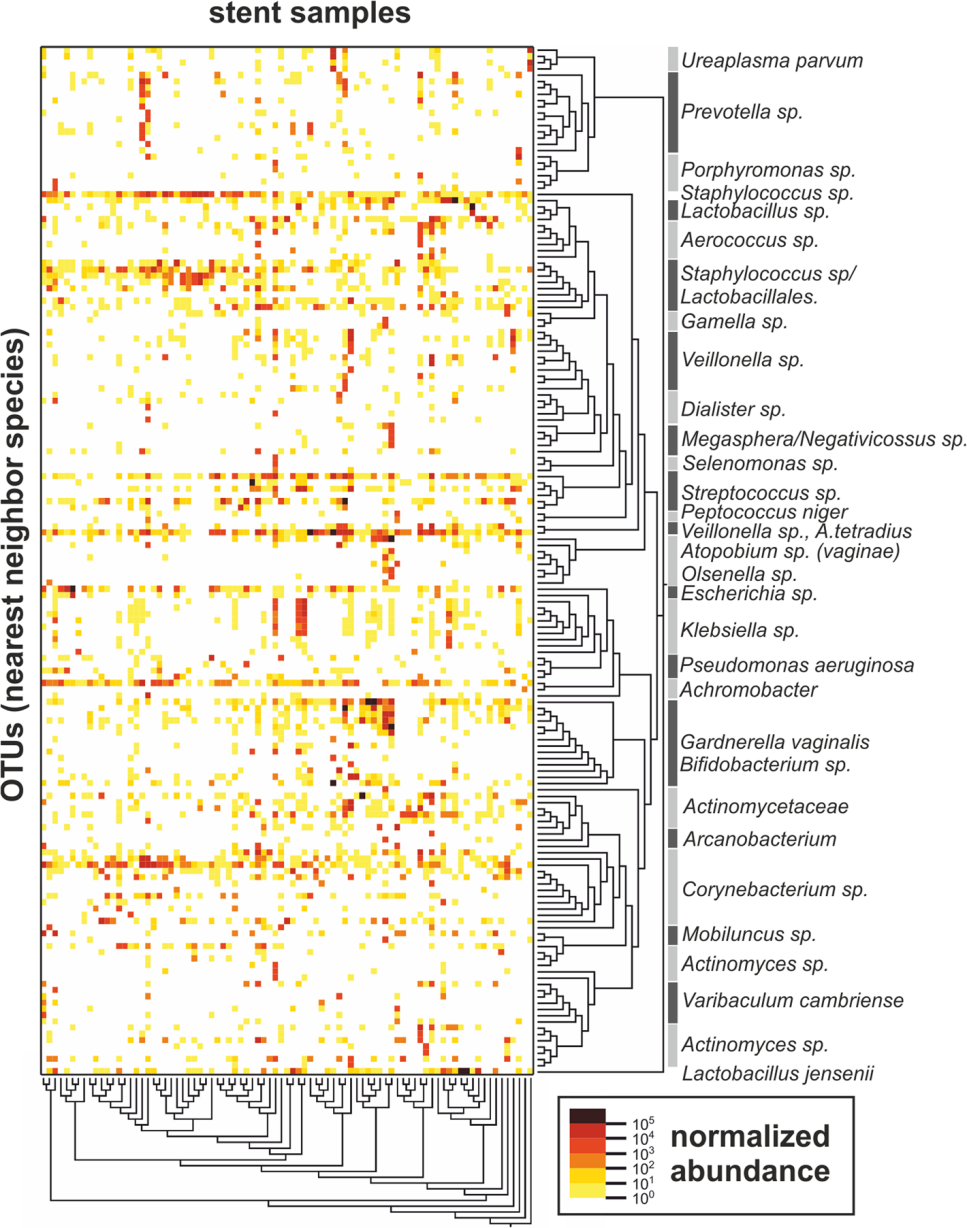
Heat map representing absolute normalized abundance of bacterial nearest neighbour species (OTUs, *y*-axis), and of ureteral stent encrustation samples (*x*-axis) ordered according to inter-sample distance. Note, horizontal dense rows of points representing OTUs found in more than 50% of the samples, likely representing commensal bacteria. The normalized abundance represents bacteria equivalents per sequencing reaction

**Fig. 5:**
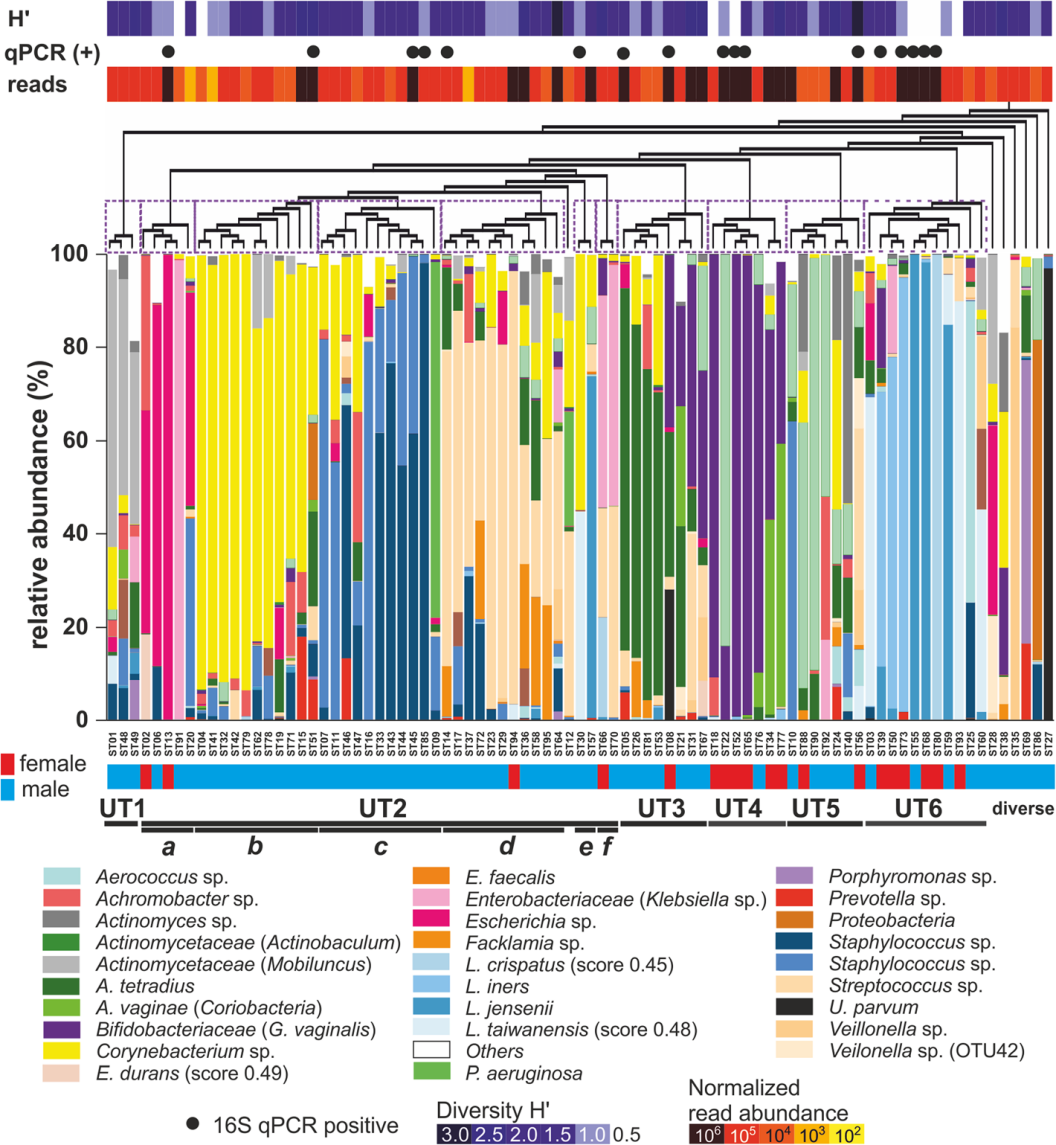
Relative abundance of 28 most abundant bacterial taxa (very similar taxa summarized under one identifier, brackets indicate abundant species or genera) in the ureteral stent encrustation samples, sorted according to inter-sample distance (beta diversity). Similar samples formed 11 clusters, termed “urotypes” (UT1–UT6, indicated by purple dashed boxes), with 6 samples of a very different community structure not being part of the clusters

To estimate the abundance of the individual OTUs detected in the samples, reads were converted into “bacteria equivalents” (bac.eq.) as a semi-quantitative unit, based on the expected 16S rRNA gene copy number of identified OTUs (“nearest-neighbour species”). Similar to the results obtained from qPCR, the NGS analysis also revealed relatively low amounts of bacteria (NGS, median 3.8 × 10^5^ bac.eq. per stent, ranging from 2620 to 6.3 × 10^6^; qPCR, median 1.3 × 10^4^ bac.eq. per stent, ranging from 86 to 4.5 × 10^7^). Bacterial abundance across the sequenced samples can be readily visualized on a heatmap (Fig. 4), in which stent samples are clustered based on read abundance and similarity as calculated with the UniFrac between-sample distance metric (beta diversity, *x*-axis) and by phylogenetic distances (*y*-axis). Thirteen OTUs (Additional file 2: Table S3) were present in varying relative amounts in more than 50% of all samples, which can be visualized on the heatmap as horizontal dense rows of points (Fig. 4). Bacterial genera and families that are commonly identified as contaminants in sequencing studies, such as *Bradyrhizobium*, *Sphingomonas* and *Ralstonia* were not found here [28]. To visualize similarities in bacterial community composition between the samples and to reduce data complexity, the 28 most abundant taxonomic groups summarized at the species, genus or family level were plotted according to relative abundances (Fig. 5). Based on the between-sample distance metric UniFrac, the samples clustered in six “urotypes” (UT), a term introduced by Pearce et al [15]. Seven samples were not assigned to any UT due to their unique or unusual microbiome profiles (i.e. ST12, ST27, ST28, ST35, ST38, ST69 and ST86). The microbiome profiles of the four patients that received antibiotic treatment during the stent indwelling period (ST09, ST24, ST76, ST93) did not feature obvious differences in their microbiome composition compared to patients without antibiotic treatment, and they remained clustered into urotypes, apart from ST09, which contained high relative percentages of *Pseudomonas aeruginosa*.

A characteristic feature for all urotypes was a high relative abundance of one to three dominant OTUs (Fig. 5, Table 2). In brief, UT1 was characterized by dominance of *Actinomycetales* (e.g. *Actinomyces*, *Actinomycetaceae*), including *Micrococcaceae* (e.g. *Staphylococcus*) and *Mobiluncus*. UT2 was divided into six sub-clusters UT2a–f, where UT2a was characterized by reduced diversity and abundance of *Enterobacterales*, particularly *Escherichia* and *Klebsiella*, mostly with only one dominant species. The solely male urotypes UT2b and UT2c were defined by an abundance of *Corynebacterium* and *Staphylococcus* in different ratios, often accompanied by *Achromobacter*. UT2d was distinguished by dominance of *Streptococcus*, frequently together with *Facklamia*, *Anaerococcus tetradius* or *Staphylococcus*; UT2e by *Lactobacillus* and *Corynebacterium*: and UT2f by *Enterobacteriaceae * (similar to or identified as *Klebsiella*; taxonomy confidence score 0.42–0.79) and *Streptococcus*. In UT3, *A. tetradius* was strongly represented*,* accompanied either by *Corynebacterium* or *G. vaginalis* and this urotype was frequently of high bacterial load. UT4, containing mostly female samples, was characterized by low diversity but high bacterial abundance, and it was dominated by *G. vaginalis*, with characteristic presence of *Atopobium vaginae* or *Actinotignum*. In the presence of *G. vaginalis*, *Corynebacterium* species were absent or strongly reduced. UT5 was characterized by abundant *Actinotignum*, while *G. vaginalis* was either absent or present in minor amounts. UT6 included *Lactobacillus*-dominated samples and was made up mainly of samples from female patients. It had a remarkably low diversity but high bacterial abundances.

**Table 2:** Characteristics of identified urotypes (groups of microbiota profiles clustered according to between-sample distance).

UT	Sample number (f/m)	Mean diversity (H’ ± SD)	Characteristic OTUs
1	3 **(0/3)**	1.80 ± 0.33	*Actinomycetales* (including the *Mobiluncus* genus and *Micrococcaceae* family)
2a*	5 (2/3)	0.84 ± 0.54	*Enterobacterales* (*Escherichia* and *Klebsiella*)
2b	11 **(0/11)**	1.27 ± 0.61	*Corynebacterium*, *Staphylococcus* and *Achromobacter*
2c	11 **(0/11)**	1.17 ± 0.36	*Staphylococcus* and *Corynebacterium*
2d	11 (1/11)	1.48 ± 0.65	*Streptococcus*, *Facklamia* and *A. tetradius*; low percentages of *Corynebacterium* and *Staphylococcus*
2e	2 **(0/2)**	1.11	*Lactobacillus* and *Corynebacterium*
2f	2 (1/1)	1.92	*Enterobacterales* and *Streptococcus*
3	8 (1/7)	1.56 ± 0.57	*A. tetradius*, *Corynebacterium* or *G. vaginalis*
4	7 (6/1)	1.02 ± 0.73	*G. vaginalis, A. vaginae* or *Actinotignum*
5**	7 (2/5)	1.67 ± 0.71	*Actinobaculum*
6	11 (6/5)	0.82 ± 0.54	*Lactobacillus*
n.a.	6 **(0/6)**	1.36 ± 0.42	diverse

Solely male specimen distribution is marked in bold print

*UT* urotypes, *f* female patient, *m* male patient, *H’* Shannon index, *n.a.* not assigned

*ST12 was excluded from UT2 due to a very different community structure based on large relative amounts of *P. aeruginosa*

**ST10 was excluded from UT5 for comparative analyses due to high amounts of *Staphylococcus* and differing community structure

In our study, solely specimen derived from male patients were allocated to urotypes 1, 2b, 2c and 2e, as well as the diverse group, and predominant male specimen made up urotypes 2d, 3 and 5. In contrast, predominantly female specimen clustered into urotypes 4 and 6.

### Crystal analysis and correlations with urotypes

The extracted amounts of encrustations and biofilms were ranging from 0–310 mg/stent (mean of 47.5 ± 46.9 mg). Only in a subset of samples (i.e. 8%) more than 100 mg were obtained (Fig. 2e). Based on the known X-ray diffraction pattern deposited in the Crystallography Open Database [32], dominant crystalline phases were found to include dicalcium phosphate dihydrate (identifier COD2310526); calcium oxalate monohydrate (whewellite, identifier COD9000763); and -dihydrate (weddellite, identifier COD9000764) (Fig. 2f), which are known to be relevant for kidney stone formation and ureteral stent encrustation [33, 34]. Samples containing dicalcium phosphate dihydrate had a significantly higher total biomass than samples with other crystalline phases (Fig. 2f) (*t* test *p* < 0.05).

**Fig. 6:**
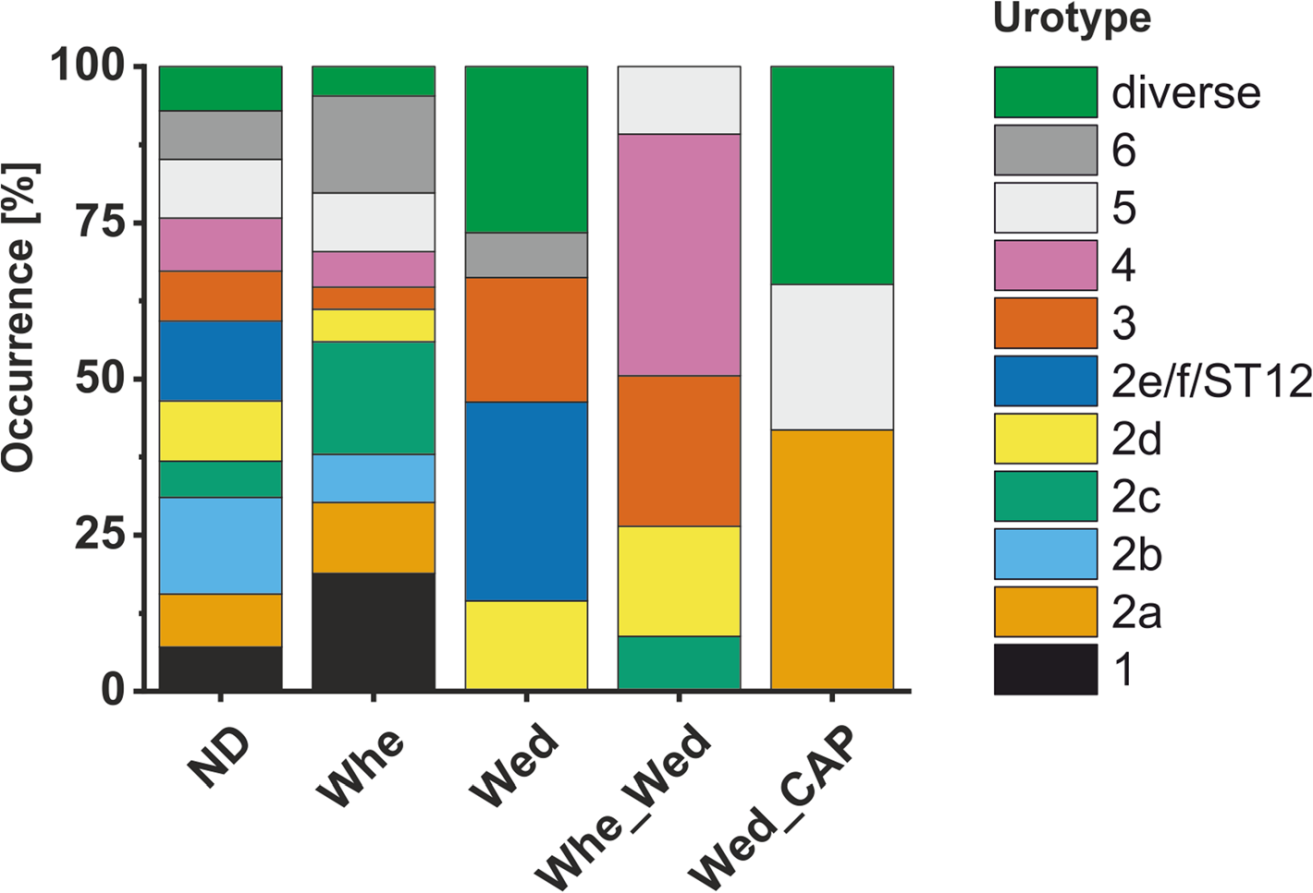
Crystal phases identified in urotypes by XRD analysis. *ND* absence of crystals, *Whe* whewellite, *Wed* weddellite, *CAP* dicalcium phosphate dihydrate

Comparison between the qualitative phase analysis of the crystalline part of the biomass and microbiome profiles revealed that whewellite was detected in almost all urotypes, except UT2e, UT2f and ST12 (Fig. 6). Dicalcium phosphate dihydrate was rarely found, only in few samples of UT2a, UT5 and the diverse group. Interestingly, in none of the urotypes UT1, UT2a, UT2b weddellite was detected. Only in a single sample of UT2c, weddellite was detected together with whewellite. In summary, XRD analysis revealed that encrustations having whewellite rather than weddellite crystals had *Enterobacterales* or *Corynebacteriaceae*-dominated microbiomes.

### Combination of cultivation and cultivation-independent analyses to identify microbiota in ureteral stent encrustations

qPCR allowed for an estimation of bacterial abundance in ureteral stent encrustations, and NGS provided information on the presence of bacterial DNA. Even though qPCR and NGS are based on a different experimental pipeline and primer sets, quantification of bacteria by qPCR correlated with NGS (Additional file 2: Figure S2b). It required combined cultivation-dependent and cultivation-independent analyses to interpret which of the identified bacteria are present in the encrustations. Apart from exceptions, the morphology of bacteria-like structures visible by SEM imaging as components of the encrustations frequently did not correspond to the bacteria revealed abundant by NGS (e.g. cocci versus rods). Cultivation of the resuspended encrustations showed bacterial growth in 35.3% (6 out of 17) qPCR-positive samples, but only for 6.9% (5 out of 72) qPCR-negative samples (chi test *p* = 0.0014). Particularly for *E. coli* or *Staphylococcus*, cultivation of urine samples and resuspended encrustations agreed with NGS (Table 3). Only for one qPCR-positive sample (i.e. ST14), no bacteria-like structures could be visualized by SEM despite cultivable bacteria.

**Table 3:** Evidence for bacteria in qPCR-positive ureteral stent encrustation samples by cultivation, NGS and SEM

UT	Sample	Dominant OTUs	Cultivation of encrustations	Urine culture (pre-surgery)	SEM	H’
2a	*ST13*	***Escherichia***	n.d.	***E. coli***	n.d.	0.56
2b	ST15*	***Corynebacterium***	***Corynebacterium***	coagulase negative *Staphylococci*, alpha haemolytic *Streptococci*, *Lactobacilli*	n.d.	2.14
	*ST51*	*Corynebacterium*	n.d.	n.d.	Coccoid	2.38
2c	*ST85*	***Staphylococcus***	***S. epidermidis***	**coagulase negative** ***Staphylococci*** *, Enterococci*	**Coccoid**	0.61
	*ST45*	***Staphylococcus***	n.d.	n.d.	**Coccoid**	1.1
2d	ST64*	***Streptococcus,*** *Klebsiella,* ***Staphylococcus***	n.d.	n.d.	**Coccoid**	2.81
	*ST14*	*Streptococcus, Facklamia,* *A. tetradius,* ***Escherichia***	***E.coli***	***E. coli***	n.d.	1.03
2e	*ST30*	***Corynebacteria,*** *Lactobacillus*	n.d.	*Corynebacteriaceae*, non-haemolytic *Streptococci*	***Coryne*** **-shaped**	0.71
3	*ST08*	*Ureaplasma parvum, A. tetradius, G. vaginalis, minor amounts* ***of Escherichia, S. anginosus***	***E.coli*** ***S. anginosus***	***E.coli*** ***S. anginosus***	Coccoid	1.83
	*ST05*	*A. tetradius, S. anginosus* *Prevotella,* ***Escherichia***	n.d.	***E. coli***	Coccoid	1.39
4	*ST22*	*Actinobaculum,* ***G. vaginalis***	*Lactobacillus iners*	***G. vaginalis***	**Rods**	0.56
	*ST52*	***G. vaginalis***	***G. vaginalis***	n.d.	Coccoid	0.40
	*ST65*	*G. vaginalis*	n.d.	*S. epidermidis*	Coccoid	1.13
5	*ST56*	*Veillonella*	n.d.	*K. pneumoniae*	Coccoid	2.59
6	*ST55*	***L. jensenii***	***L. jensenii***	***L. jensenii***	**Rods**	0.03
	*ST80*	*L. crispatus*	n.d.	*Streptococcus gallolyticus*	n.d.	0.32
	*ST73*	*L. iners*	n.d.	*Corynbacterium amycolatum, S. epidermidis*	Coccoid	0.96
	ST39	*L. iners*	n.d.	*Aerococcus urinae, Aerococcus sanguinicola, S. epidermidis, S. aureus*	n.d.	1.50
	ST68	*L jensenii*	n.d.	*Corynebacteriaceae,* coagulase negative *Staphylococci*, alpha haemolytic *Streptococci*, *Lactobacilli*	n.d.	0.19

Boldface data denote concordant results of NGS and cultivation of the encrustations, urine culture or SEM.

*samples very close to the LOD of the qPCR assay

*n.d.* not detected, *H’* Shannon index

As part of urine analysis, urine samples were tested for presence of nitrate-reducing bacteria. In five specimen (i.e. ST2, 8, 13, 19, 48) patients that did not have detectable urine nitrite amounts before stent placement, detectable nitrite was found in urine analyses before stent removal. Vice versa, only one specimen showed detectable amounts of nitrite before stenting but not before stent removal (i.e. ST44), and one specimen revealed nitrate before stent placement and stent removal.

A particularly high bacterial load was found for samples of the *Lactobacillus*-dominated UT6, such as ST55, which contained visible *Lactobacillus*-shaped bacteria-like structures and high absolute and relative abundance of *L. jensenii* in NGS, which was further confirmed by cultivation-based detection of *Lactobacillus*. In contrast, putative *L. crispatus* (score 0.45) as well as *L. iners* seemed not to be part of the encrustations and lacked cultivable or visible bacteria, although NGS and qPCR indicated high amounts of respective bacterial DNA (Table 3).

Samples with *G. vaginalis*-dominated urotypes (UT3 and UT4) frequently contained high amounts of bacterial DNA, but encrustation-associated structures with rod-shaped morphology, similar to *G. vaginalis*, could rarely be visualized. In ST22, for example, NGS indicated high absolute amounts of *Actinobaculum* and *G. vaginalis*, with short rod-shaped bacteria visible by SEM. Cultivation of the suspended encrustations revealed high numbers of *G. vaginalis*, the second most abundant strain according to NGS. Similarly, ST52 and ST65 were dominated by *G. vaginalis* which was also detected by cultivation of encrustations from ST52, but imaging revealed only coccoid bacteria-like structures. ST08 (UT3) was dominated by *U. parvum, A. tetradius* and *G. vaginalis*, with minor relative contribution of *Escherichia* and *Streptococcus*, but only coccoid-shaped cells were found by imaging. Cultivation of both urine and encrustations, in contrast, revealed growth of *E. coli* and *S. anginosus*.

*Escherichia* (i.e. *E. coli*) are typical urinary tract pathogens, capable of adhering to surfaces and forming biofilms. For the *Enterobacterales-*dominated sample ST13, however, neither did imaging reveal bacteria nor could bacteria be cultivated from resuspended encrustations. Urine culture before stent removal, however, revealed growth of *E. coli*, indicating presence of *E. coli* in the bladder, but not in the encrustations. Importantly, it needs to be noted that no substances inhibiting bacterial growth, such as antibiotics, were found in resuspended encrustations of this sample. Overall, for pre-operative urine culture, as well as resuspended encrustation cultivation, no growth-inhibiting substances have been detected, apart for ST02 (growth of *Candida glabrata*) and ST09 for pre-surgery urine, and ST49 and ST27 for resuspended encrustation (data not shown).

Sample ST45 (UT2c) was dominated by *Staphylococcus*, but SEM revealed only a few bacteria-like structures, and no bacteria could be cultivated from the encrustations or urine. In contrast, abundant bacteria could be visualized for the *Staphylococcus epidermidis*-dominated ST85, which was also identified by urine culture and culture of the encrustations.

Among *Corynebacterium*-dominated samples ST15 and ST51 (UT2b), coccoid bacteria were found by microscopy for ST51, while cultivation of encrustations yielded growth of *Corynebacterium* for ST51. Only for one sample of UT2e (ST30), a patch of club-shaped bacteria could be visualized, indicative for *Corynebacterium*, accompanied by few cocci, while no bacteria could be cultivated from the encrustations (Additional file 2: Figure S1).

## Discussion

This work provides insights into the microbiome of ureteral stent encrustations and information on the bacterial contribution to these encrustations for the first time. In contrast to microbiome studies investigating samples of high bacterial content, the low bacterial load in urinary tract samples complicates the generation of accurate microbiota profiles. Accordingly, contaminations from reagents or sample processing may mask the signal of resident microbiota [35]. Moreover, PCR-inhibitors and human DNA transrenally excreted to the urine may complicate the detection of bacteria [27]. Furthermore, it can be anticipated that a certain proportion of bacterial DNA detected in urine or the urinary tract derives from extracellular DNA (eDNA) of degraded bacterial cells anywhere in the body, since eDNA is able to pass the kidney barrier in healthy humans [36–38]. However, the adsorption of eDNA to crystals and polymers may reduce the efficiency of the DNase digestion, as it was demonstrated for example for eDNA adsorbed to graphene oxide [39]. In this study, no OTUs were repeatedly identified in all samples or the majority (> 90%) of the samples, which, based on their frequency of detection, might derive from contamination. Also, common contaminants [35] were not detected. This creates good confidence that the identified bacteria originate from the urinary tract of the patients.

The use of complementary methods including sequencing, qPCR, cultivation and microscopy indicates that the investigated encrustations mostly contained very few bacteria. Quantification of 16S rRNA gene copy numbers by qPCR showed that only about one fifth of the samples contained bacterial DNA above the LOD. Cross-evaluation with microscopy revealed that qPCR-based detection of more than 2 × 10^5^ bacteria equivalents per stent gave good confidence for the presence of (visible) bacteria in the encrustations. Overall, high amounts of bacterial DNA corresponded with visible or cultivable bacteria. However, the technical limitations of microscopy need to be considered since bacteria may not be visible when growing patchy outside of the investigated areas, or when covered by extracellular polymeric substances.

Most encrustations consisted predominantly of deposited urine components such as inorganic crystalline material or polymers, while bacteria were visible only in a subset of the samples. The observed low bacterial load and rare occurrence of heavily colonized ureteral stent encrustations may be partially attributed to the investigated study cohort of exclusively stone-forming patients who had no bacteriuria (i.e. < 10^4^ mL^-1^ detected bacteria by standard methods). Furthermore, the stent indwelling time of 3–6 weeks was relatively short. Therefore, an analysis of long-term inserted ureteral stents (e.g. up to 6 months), including those from cancer patients, may reveal a much higher bacterial load.

Since the identified microbiome profiles were highly similar to urinary microbiomes described previously [15, 16, 18], we speculate that a substantial portion of the DNA found in the encrustations likely derive from bladder microbiota and deposit over time. The herein identified microbiome profiles may represent the cumulated microbiome over stent indwelling time, through continuous adsorption of eDNA to the encrustations.

Even though sorting of the samples into urotypes provided important insights into the urinary tract microbiome, it needs to be considered that all related findings are subjected to technical limitations. PCR-based detection of bacteria depends on primer sets, PCR bias related to cycle number, polymerase specificity and the amplified region of the selected phylogenetic marker [40, 41]. Furthermore, categorizing microbiome data according to phylogenetic groups, or clustering of samples, has limitations since the species composition may be better represented in gradients rather than distinct groups, although it helps with the classification of patient samples [42]. The categories of urotypes likely represent the extremes of continuous gradients, similar to the human gut microbiome [43].

To identify associations of microbiome profiles (i.e. urotypes) with different patient conditions, urotypes were compared with patient characteristics and laboratory findings, including age, gender, body mass index, diabetes, weight, patient’s morbidity (ASA score, physical status classification system American Society of Anaesthesiologists), extracted biomass, blood and urine leukocytes and erythrocytes or crystalline components in the encrustations and biofilms. Correlations were generally weak (*data not shown*). For almost all patients (except for four), urine leukocytes and erythrocytes drastically increased during stenting (data not shown), which is known to cause mechanical irritation of the urothelial cell layer [44].

The patients included into this work have further been subject of a clinical study, analysing correlations between ureteral stent symptoms, as assessed by a questionnaire, which revealed a significant correlation between bacterial load and patient’s pain and intake of analgetics, as well as a correlation between the mass of the encrustation and hematuria (Betschart et al. 2018). Between the individual urotypes and these parameters, however, no correlations have been found (*data not shown*).

Many microbiome profiles were dominated by bacteria known to be prevalent in women with bacterial vaginosis, such as *Mobiluncus* [45], *Prevotella* [46] or *G. vaginalis* and *A. vaginae* [15, 18, 47] but are also found in asymptomatic patients [48]. Similarly, many samples contained significant relative amounts of *Enterobacterales*, which are commonly recognized as uropathogens, with *E. coli* being detected by standard cultivation methods in clinical diagnostic laboratories in the great majority of uncomplicated UTIs. *E. coli* may dominate the urinary microbiome but remain asymptomatic [49]. The presence of a potentially pathogenic strain alone, however, does neither determine a clinically manifested infection nor does it represent a higher risk for infection or result in an induced immune response. Since this study investigated exclusively patients not suffering from infectious complications or respective symptoms before or at the time of stent insertion, the observed bacteria can be regarded as commensal microbiota of healthy patients.

Microbiome profiles dominated by *Lactobacillus* and *Corynebacterium* are likely to be associated with health*.* Most *Lactobacillus* species are regarded as beneficial bacteria, as they are known to establish their ecological niche by mechanisms such as competitive exclusion, secretion of antimicrobial peptides, biosurfactants or modulation of the host immune system [50]. Accordingly, in our study, *Lactobacillus*-dominated microbiome profiles were of very low diversity. Clinical trials have proven some efficiency for *L. crispatus* in the reduction of recurrent UTIs [51], while *L. jensenii* inhibits *Neisseria gonorrhoeae* in vitro [52]. Further, in *L. crispatus-*dominated vaginal swab samples, disease-associated pathogens were rarely identified, and this strain was found associated with lack of urgency urinary incontinence syndrome symptoms [15, 18]. *L. iners*, instead, was also found in individuals with bacterial vaginosis, and it is associated with an increased susceptibility to *Chlamydia trachomatis* infection [53, 54]. In this study, *L. iners* was found accompanied by *Enterobacterales.* Since the investigated patients did not have a UTI, no conclusions can be made on beneficial effects of this strain on UTI.

While the *Lactobacillus-*dominated urotype 6 was predominantly found for female patients, *Corynebacteriaceae* and *Staphylococcus*-dominated urotypes 2b and 2c were exclusively detected for male patients*. Corynebacteriaceae* have been reported as the normal urogenital microbiota of healthy men [2]. However, many microbiome profiles had abundant amounts of *Corynebacterium tuberculostearicum*, which may be also involved in infections [55]. Only in one sample (ST30), *Corynebacteriaceae-*shaped cells were identified as part of the encrustations. In this work, no strong associations between health condition and microbiota were identified.

*Corynebacteriaceae*-dominated microbiomes tended towards a different crystal composition with absence of weddellite (calcium oxalate dihydrate). The tetragonal-dipyramidal crystal structure of weddellite has visibly (see Fig. 2c) sharp edges which may contribute to irritation and vulneration of the urothelial cell layer. Evidence for such an association between distinct microbiome profiles and kidney stone formation has been found for the gut microbiome [56].

## Conclusions

This is the first study to present cultivation-independent information on the ureteral stent microbiome. A combination of sequencing, qPCR and imaging led to the conclusion that ureteral stent encrustations of patients in absence of UTI or bacteriuria (i.e. > 10^4^ mL^-1^ detected bacteria by standard methods) have a low bacterial load and mostly consist of deposited urine components and crystals grown on the surface of the biomaterial in case of short-term stenting. The patients could be classified in different urotypes, which were similar to urotypes previously identified in the urinary microbiome [15, 18]. Several samples were dominated by bacteria which are known as facultative pathogens which, however, appear to be a common feature in patients without clinically manifested UTI. The diversity of *Lactobacillus*-dominated microbiomes was strongly reduced and may represent a suppression of facultative pathogenic bacteria by typical *Lactobacillus*-derived antimicrobials. An improved understanding of the commensal relationships in urinary tract microbiome and factors influencing their balance might help with defining a healthy microbiome and finding treatments without disrupting the healthy microbiome.

## Methods

### Aim of the study and study design

This study aimed for a better understanding of the ureteral stent biofilm microbiome, including insight into the bacterial load. Investigated ureteral stent biofilms derived from 89 patients who underwent ureteral stenting due to urinary calculi for a duration of 3–6 weeks. Included patients had no UTI or positive urine cultures when entering the study. Patients with bilateral stenting, stenting due to malignant tumours and those who were subjected to additional surgical procedures during stenting were excluded from the study. In accordance with urological guidelines [57], perioperative antibiotic prophylaxis was administered to the patients 1 h before removal of the stent using a single-dose trimethoprim/sulfamethoxazole 160/800 mg/os. The clinical trial was registered under the identifier “NCT02845726” at clinicaltricals.gov, where further study details can be found [58]. The patient’s blood and urine were examined before stent application and removal, including urine sediment. Using routine cultivation techniques, urine samples and resuspended encrustations were tested for cultivable bacteria. Sections of the tips of the ureteral stents were analysed by SEM for bacteria-shaped structures, extracted encrustations were balanced and analysed by XRD for calcium oxalate or calcium phosphate phases, and the extracted DNA was subjected to qPCR and NGS.

### Sample collection, extraction of encrustations and DNA

For the removal of ureteral stents, the bladder was entered with the sterile cystoscope after topical disinfection of the external genitalia with 0.1% octenidine solution. The bladder was emptied through the cystoscope and filled with sterile saline solution, followed by removal of the ureteral stents through the outer shaft of the cystoscope. Removed stent samples were cut with sterile scissors in the middle and stored in ethylene-oxide-treated polypropylene tubes humidified with 2 mL DNA-free physiological saline solution for a maximum of 4 h at 6 °C until extraction of the encrustations. After taking samples for SEM imaging, encrustations were extracted by mechanical abrasion using DNA-free equipment and techniques, as previously described [59]. The stent lumen was excluded from the analyses as in our hands, it was not possible to be extracted using DNA-free techniques. Ten percent of the 1-ml resuspended encrustations was then transported to the clinical diagnostic laboratory for cultivation-dependent analyses, and 20 μL of the suspension were kept for later XRD analyses. DNA from resuspended encrustations was extracted under a laminar flow workbench using a DNA-free plastic ware and the Molzym Complete5 DNA extraction kit (Molzym, Germany), including DNase digestion of extracellular DNA [60]. To exclude the introduction of contaminating DNA during the sampling pipeline, potential DNA contamination from three blind samples was extracted, unused ureteral stents that were unpacked in the surgery cabinet and subsequently treated analogous to the clinical samples.

### Microscopic analyses

SEM was performed as described previously [59]. In brief, ureteral stents sections of 2 mm were cut with a flame-sterilized DNA-free scalpel on a flame-sterilized glass surface at the proximal and distal loop ends, fixed for 30 min in glutaraldehyde and formaldehyde and stored in 0.9% saline solution at 4 °C until dehydration using a series of increasing ethanol concentrations. Dry stent sections were treated with 1,1,1-trimethyl-N-(trimethylsilyl)silanamine and dried in a fume hood prior to sputtering with 7 nm Au/Pd in an EM ACE600 machine (Leica, Switzerland) and analysed with an S-4800 scanning electron microscope (Hitachi, Japan) at 2 kV acceleration voltage. Bacteria were defined as identified by microscopy when more than three structures in the size and shape of bacteria were identified on the images. Of each sample, multiple images of different positions of the stent surface and lumen were taken.

CLSM was performed on formaldehyde-fixed samples that had been stored in phosphate-buffered saline (PBS). From the samples, 2–3-mm sections were cut with a scalpel and mounted in 2.5-mm microscopy chambers (Cat# 70327-25; 2.5-mm depth, 20-mm diameter, Electron Microscopy Sciences, emsdiasum.com). For staining, a SybrGreen 1 solution in PBS was added according to the manufacturer’s instructions. Finally, the chambers were filled up with PBS and covered with a 25 × 25-mm coverslip. Overflowing liquid was removed with small pieces of Whatman paper. Microscopy was performed with a TCS SP5X AOBS confocal laser scanning microscope (Leica, Germany), controlled by the LSAF Software version 2.4.1 build 6384. The system was equipped with an upright microscope and a super continuum light source (470–670 nm) as well as a 405-nm pulsed laser diode. Images were collected with a 25-fold water immersion lens with a numerical aperture (NA) of 0.95. Excitation was at 494 nm with 75% laser intensity (instrument) and 50% (software). Emission was recorded in the range of 489–499 nm (reflection) and 505–560 nm (fluorescence).

### Crystal analysis

A small fraction (i.e. 5%) of resuspended ureteral stent encrustation samples was screened by powder XRD for the presence of dominant crystalline phases. The samples corresponding to 20 μL resuspended sonicated encrustations fixed in 4% formaldehyde were dried, homogenized, suspended in carrier oil (HRZ-643 Parabar 10312) and placed into the beam to allow the X-rays to pass through the sample; a transmission mode was used. Qualitative phase analysis of resuspended ureteral stent encrustation samples was performed by two-dimensional wide-angle XRD (2D-WAXD) analysis using a STOE IPDS-II instrument (Stoe & Cie GmbH, Germany). WAXD patterns were recorded using Mo Kα radiation (*λ* = 0.71073 Å) at 40 mA and 50 kV. The samples were exposed for 5–30 min. In a stream of liquid nitrogen at a controlled temperature of − 100 °C, the WAXD patterns were recorded on an image plate detector system with a 340-mm diameter placed at a distance of 200 mm from the sample. 2D images have been recorded for all investigated samples covering a 2 theta range from 1.5° to 40°. Intensities were integrated (360°) and analysed using the DIFFRAC.EVA software version V4.3 (Bruker, Germany) by comparison with the COD reference database [32]. The presence of the following crystallographic phases has been found: calcium oxalate monohydrate, whewellite (C_4_H_2.57_ Ca_2_O_10_ COD-ID 9000763), calcium oxide dihydrate, weddellite (C_2_H_6_CaO_6.375_; COD 9000763) and dicalcium phosphate dehydrate (CaH_5_O_6_P; COD 2310526). Further details on the indexing of the crystalline phases can be found in Additional file 2: Figure S5.

### Sequencing library preparation

First-step PCR amplification was done using non-diluted, ten and hundred times diluted template with the S-D-Bact-0341-b-S-17 and S-D-Bact-0785-a-A-21 primer pair with Illumina adapters [41] (1st_PCR_for_S-D-Bact-0341-b-S-17: NGS_1st_PCR_Fwd TCGTCGGCAGCGTCAGATGTGTATAAGAGACAGNNNNNNNNCCTAC-GGGNGGCWGCAG) and 1st_PCR_rev_ S-D-Bact-0785-a-A-21: NGS_1st_PCR_Rev GTCTCGTGGGCTCGGAGATGTGTATAAGAGACAGNNNNNNNNGACTACHVGGG-TATCTAATCC), using the KAPA HIFI Taq HotStart PCR kit according to the manufacturer’s instructions. Three PCR reactions each with either 10 μL template used non-diluted, ten- or 1000-fold diluted, were performed on a CFX96 thermocycler (BioRad, Germany), with initial denaturation for 5 min at 95 °C and 34 cycles of 30 s at 95 °C, 30 s annealing at 60 °C and 30 s elongation at 72 °C. After a final elongation step for 30 s at 72 °C, reactions were cooled down to 8 °C. Ramp rates were set to 1.4 °C s^-1^. 1.2% agarose gel electrophoresis revealed specific amplification and absence of bands for water controls. The three PCR reaction products were pooled and column-purified (GeneJet DNA Cleanup Micro Kit, Thermo Scientific). Eluates were diluted to 30 μL and stored in a 96-well PCR plate at − 80 °C.

### Illumina MiSeq sequencing, amplicon-metagenomics data processing and statistical analyses

To sequence the V3–V4 region of the bacterial 16S rRNA gene, two-step Nextera PCR libraries were sequenced using the Illumina MiSeq platform and a v2 500 cycles kit. The produced paired-end reads which passed Illumina’s chastity filter were subjected to de-multiplexing and trimming of Illumina adaptor residuals using Illumina’s real-time analysis software version 1.18.54 (no further refinement or selection). The quality of the reads was checked with the software FastQC version 0.11.5 [61]. The locus-specific V3–V4 primers were trimmed from the sequencing reads with the software Cutadapt v1.14 [62]. Paired-end reads were discarded if the primers could not be trimmed. Trimmed forward and reverse reads of each paired-end read were merged to in silico reform the sequenced molecule considering a minimum overlap of 15 bases using the software USEARCH version 10.0.240 [63]. Merged sequences were then quality-filtered allowing a maximum of one expected error per merged read and also discarding those containing ambiguous bases. The remaining reads were denoised using the UNOISE algorithm implemented in USEARCH to form OTUs, discarding singletons and chimeras in the process [64]. OTUs were compared with the reference sequences of the RDP 16S database [65], and taxonomies were predicted considering a minimum confidence threshold of 0.7 using the SINTAX algorithm implemented in USEARCH [66]. Alpha diversity was estimated using the Richness (observed), Chao1 and Shannon indices. Beta diversity was calculated using the UniFrac distance method on the basis of normalized OTU abundance counts per sample. For the visualization of relative abundances, taxonomic groups were summarized according to phylogenetic distances. Alpha and beta diversity calculations and the rarefaction analysis were performed with the software Phyloseq v1.16.2 [67]. To detect differentially abundant OTUs depending on collected patient metadata (e.g. gender) differential OTU analysis on normalized abundance counts was performed with the software DESeq2 v1.12.4 [68].

Boxplots, heatmaps and bar charts were created with the Origin software version 2018G (OriginLab, USA). In boxplots, maxima and minima are represented by horizontal lines, 1 and 99% by crosses, the mean by a black box. Boxes represent 25 and 75% quartiles, the horizontal line the data median. Pearson correlation for the DNA standard curve, significant differences for PCR samples with the Mann-Whitney test, *t* test and Kruskal-Wallis correlation between urotypes were calculated with the GraphPad Prism version 6.07 (GraphPad Software, USA), chi test with Microsoft Excel.

### Quantification of bacterial 16S rRNA gene copies by qPCR

Quantification of bacterial DNA by amplification of the 16S rRNA gene via qPCR was performed as previously described [59], using recombinant *E. coli* 16S rRNA gene plasmid DNA standard on each PCR plate for inter-run calibration. Seven no template controls were run per PCR plate, and four recombinant DNA standards. Extracted DNA was diluted 100-fold to sufficiently dilute out PCR inhibitors, as controlled in reactions using recombinant plasmid DNA containing the *E. coli* 16S rRNA gene as internal standard (*not shown*). The LOD was calculated according to the IUPAC 3 sigma criterion [69].

### Cultivation-dependent analyses and urine analysis

Ureteral stent encrustations resuspended in 0.9% saline solution were stored on ice and transported to the clinical microbiology laboratory. The suspensions were cultivated following established protocols in our routine laboratory (ISO/IEC 17025 quality assurance).

On the day of stent placement and before stent removal, midstream urine samples of male patients were collected after cleansing with saline. Intermittent bladder catheterization was performed for women after topical disinfection with povidone-iodine (Betadine®) solution or 0.1% octenidine solution (Octenisept®). If automated urinary sediment analyses revealed epithelial cells > 20/μL, sample collection was repeated.

By standard, 10 μL of suspended encrustations or urine sample was used to inoculate a Columbia agar dish (“sheep blood” agar) and a BD BBL™ CHROMagar™ Orientation agar dish (Becton Dickinson, USA). Analytical sensitivity was therefore < 1000 colony forming units (CFU) mL^-1^. Immediately after streaking, media were transferred to incubators with 36 °C aerobic (CHROMagar™ Orientation agar) or CO_2_-enriched (Columbia agar) atmosphere. In case Columbia agar was negative (i.e. no growth) after 19 h, incubation was prolonged by 1 day to ensure growth and detection of slow-growing bacteria like *Corynebacterium*. Based on the bacterial growth on Columbia agar, semi-quantitative quantification of growth was done, extrapolated to 1 mL (e.g. < 1,000 CFU mL^-1^, 10^4^ CFU mL^-1^, 10^5^ CFU mL^-1^ and ≥ 10^6^ CFU mL^-1^). With the exception of *E. coli*, which shows typical growth characteristics allowing direct identification straight from the CHROMagar™ Orientation agar, identification was done with MALDI-ToF (matrix-assisted laser desorption ionization-time of flight) mass spectrometry. Colonies were picked with a toothpick and transferred to a target, covered with matrix (i.e. direct smear method, or—if needed—with the addition of formic acid) and analysed with a MALDI Biotyper instrument and the latest version of spectrum database (Bruker Daltonics, Germany). The scoring recommended by the manufacturer was applied to finally assign a species identification.

Urine samples and resuspended encrustations were tested for substances inhibiting bacterial growth according to standard protocols [70]. Ten microlitres were dropped on a blank disc and placed on a MEDCO inhibition agar containing spores of *Bacillus subtilis* BGA (Axonlab, Baden-Dättwil, CH). Following 20–24 h of incubation at 30 °C and sporulation of the *B. subtilis*, any inhibition zone in the *B. subtilis* lawn around the disc was recorded as inhibition by urine/encrustration extract.

## Additional files

Additional file 1: Detailed patient and sample characteristics and relevant clinical data. (XLSX 66 kb)

Additional file 2: **Figure S1.** Additional SEM images representing the presence of fungi, coverage by EPS and bacterial load. **Figure S2.** a Standard curve of serially diluted recDNA containing the *E. coli* 16S rRNA gene (data from 14 independent experiments). The 16S rRNA gene copy number was plotted against the calculated gene copy number (slope = 1.03011 ± 0.07591, intercept − 0.16974 ± 0.59028, *R*^2^ = 0.98397, *n* = 14). Inter-run calibration was performed using the recDNA. b Bacteria equivalents assessed by NGS plotted vs. 16S rRNA gene copies as assessed by qPCR. 16S qPCR correlated with normalized NGS reads (Spearman ranks *r* = 0.620, *p* < 0.0001, 95% CI 0.4642 to 0.7390). **Table S3**. OTUs present in more than 50% of the samples. **Figure S4.** Rarefaction curves of the 86 sequenced samples. **Figure S5.** XRD signals and comparison with expected patterns from the COD database. (DOCX 2772 kb)
