# Supplementary material for: Encrustations on ureteral stents from patients without urinary tract infection reveal distinct urotypes and a low bacterial load
Source: Microbiome. 2019 Apr 13;7:60. doi: 10.1186/s40168-019-0674-x (PMC6462311; doi:10.1186/s40168-019-0674-x)
Supplement: Supplementary file 2 — Figure S1. Additional SEM images representing the presence of fungi, coverage by EPS and bacterial load. Figure S2. a Standard curve of serially diluted recDNA containing the E. coli 16S rRNA gene (data from 14 independent experiments). The 16S rRNA gene copy number was plotted against the calculated gene copy number (slope = 1.03011 ± 0.07591, intercept − 0.16974 ± 0.59028, R2 = 0.98397, n = 14). Inter-run calibration was performed using the recDNA. b Bacteria equivalents assessed by NGS plotted vs. 16S rRNA gene copies as assessed by qPCR. 16S qPCR correlated with normalized NGS reads (Spearman ranks r = 0.620, p < 0.0001, 95% CI 0.4642 to 0.7390). Table S3. OTUs present in more than 50% of the samples. Figure S4. Rarefaction curves of the 86 sequenced samples. Figure S5. XRD signals and comparison with expected patterns from the COD database. (DOCX 2772 kb) [file 40168_2019_674_MOESM2_ESM.docx]

Supplementary Information

**Quantitative Analysis of the Ureteral Stent Biofilm Microbiome reveals Distinct Urotypes and a Low Bacterial Load**

Matthias T. Buhmann, Dominik Abt, Oliver Nolte, Thomas R. Neu, Sebastian Strempel, Werner C. Albrich, Patrick Betschart, Valentin Zumstein, Antonia Neels, Katharina Maniura-Weber, Qun Ren

| 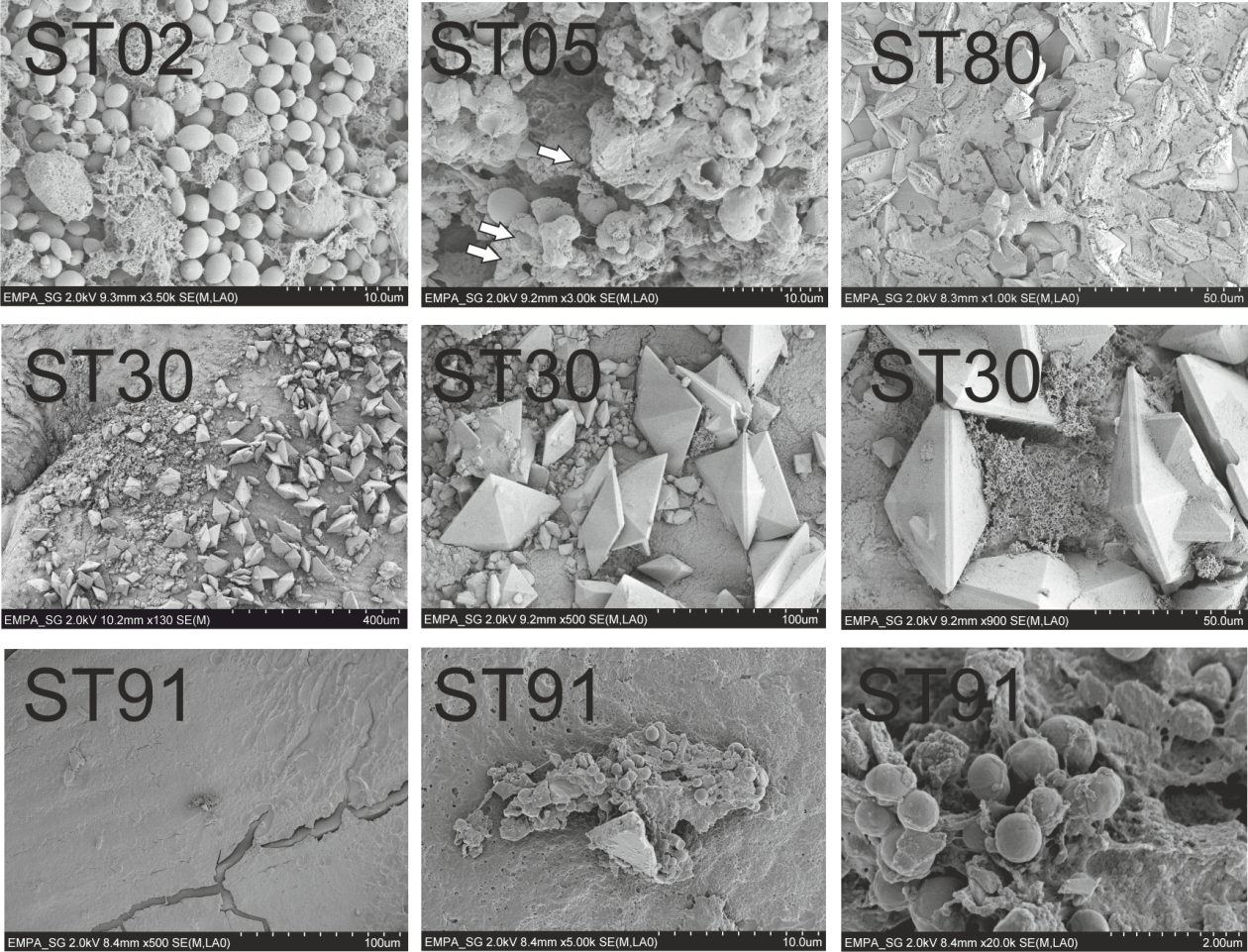 |
| --- |
| **Figure S1** Additional SEM images representing presence of fungi, coverage by EPS and bacterial load. **ST02** structures likely representing fungal cells, presumably *Candida glabrata* according to cultivation; **ST05** large presumably blood cells and few bacteria indicated by arrows; **ST80** polymers covering the crystal surface; **ST30** bacterial biofilm growing between crystals; **ST91** different magnifications of a single attached bacterial aggregate. |

| 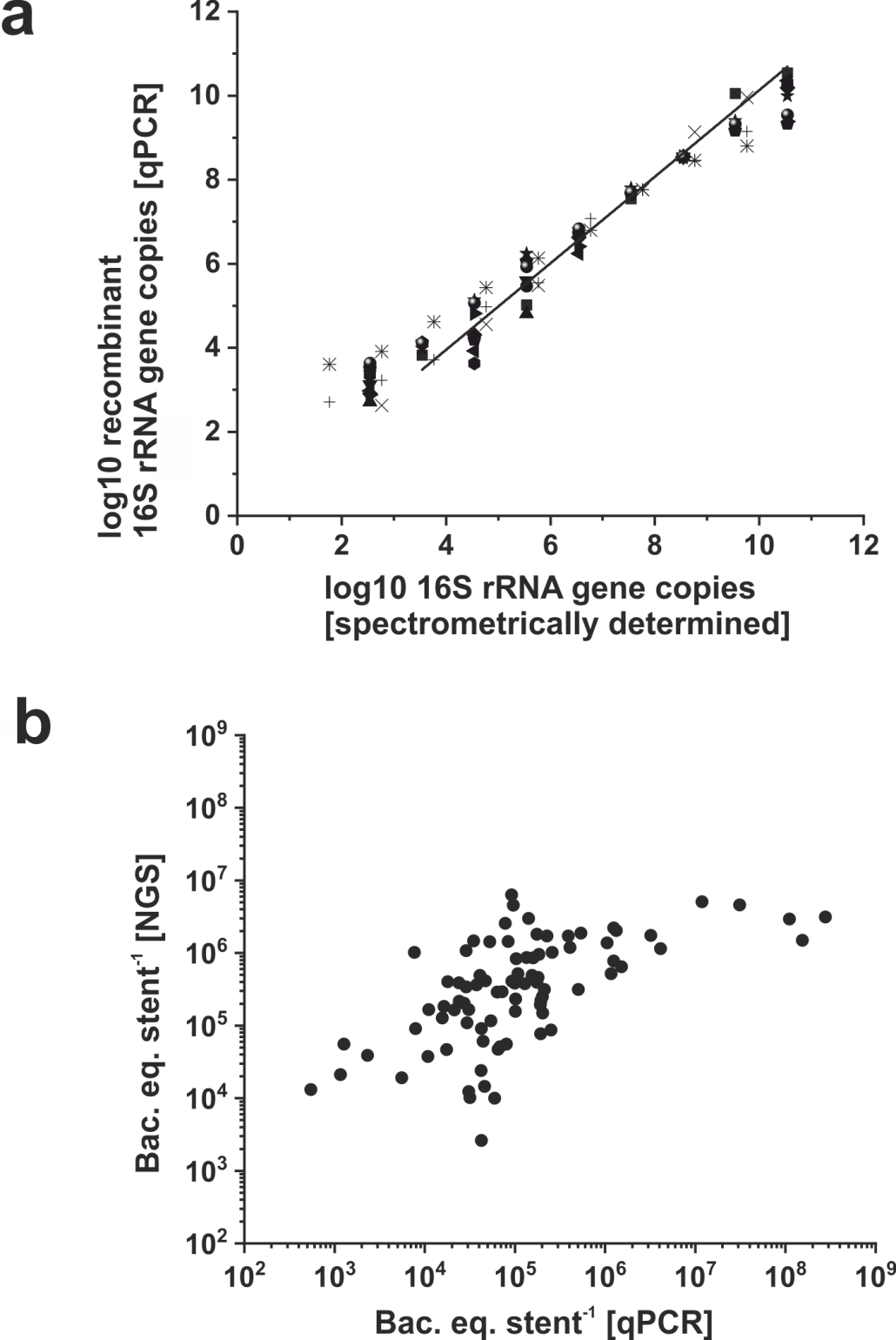 |
| --- |
| **Figure S2** **a** Standard curve of serially diluted recDNA containing the *E. coli* 16S rRNA gene. (data from 14 independent experiments). The 16S rRNA gene copy number was plotted against the calculated gene copy number (slope= 1.03011 ± 0.07591, intercept -0.16974 ± 0.59028, R^2^= 0.98397, n=14). Inter-run calibration was performed using the recDNA. **b** Bacteria equivalents assessed by NGS plotted vs. 16S rRNA gene copies as assessed by qPCR. 16S qPCR correlated with normalized NGS reads (Spearman Ranks *r*=0.620, *p*<0.0001, 95% CI 0.4642 to 0.7390). |

**Table S3.** OTUs present in more than 50 per cent of the samples.

|  | **Count** | **% of samples** | **Nearest neighbor species (score)** |
| --- | --- | --- | --- |
| OTU1 | 44 | 51.8 | *Lactobacillus jensenii* (1.0000) |
| OTU2 | 50 | 58.8 | *Escherichia fergusonii* (0.9800) |
| OTU3 | 47 | 55.3 | *Gardnerella vaginalis* (0.9900) |
| OTU4 | 74 | 87.1 | g:*Staphylococcus* (1.0000), s:*Staphylococcus* *capitis* subsp. *capitis*(0.5300) |
| OTU5 | 48 | 56.5 | *Lactobacillus iners* (1.0000) |
| OTU8 | 63 | 74.1 | *Streptococcus* *anginosus* subsp. *whileyi* (0.9700) |
| OTU15 | 45 | 52.9 | *Corynebacterium* *pyruviciproducens* (0.9800) |
| OTU16 | 64 | 75.3 | *Corynebacterium* *tuberculostearicum* (1.0000) |
| OTU19 | 60 | 70.6 | *Staphylococcus* *capitis* subsp. *capitis* (0.3000) |
| OTU22 | 43 | 50.6 | *Streptococcus* *dentisani* (0.8300) |
| OTU24 | 43 | 50.6 | o:*Lactobacillales* (0.9800), f:*Aerococcaceae* (0.9600), g:*Facklamia* (0.9000), s:*Facklamia hominis* (0.6700) |
| OTU40 | 64 | 75.3 | g:*Achromobacter* (1.0000), s:*Achromobacter spiritinus* (0.7900) |
| OTU291 | 75 | 88.2 | *Anaerococcus tetradius* (0.9800) |

*o* order*, f* family*, g* genus, *s* species

| 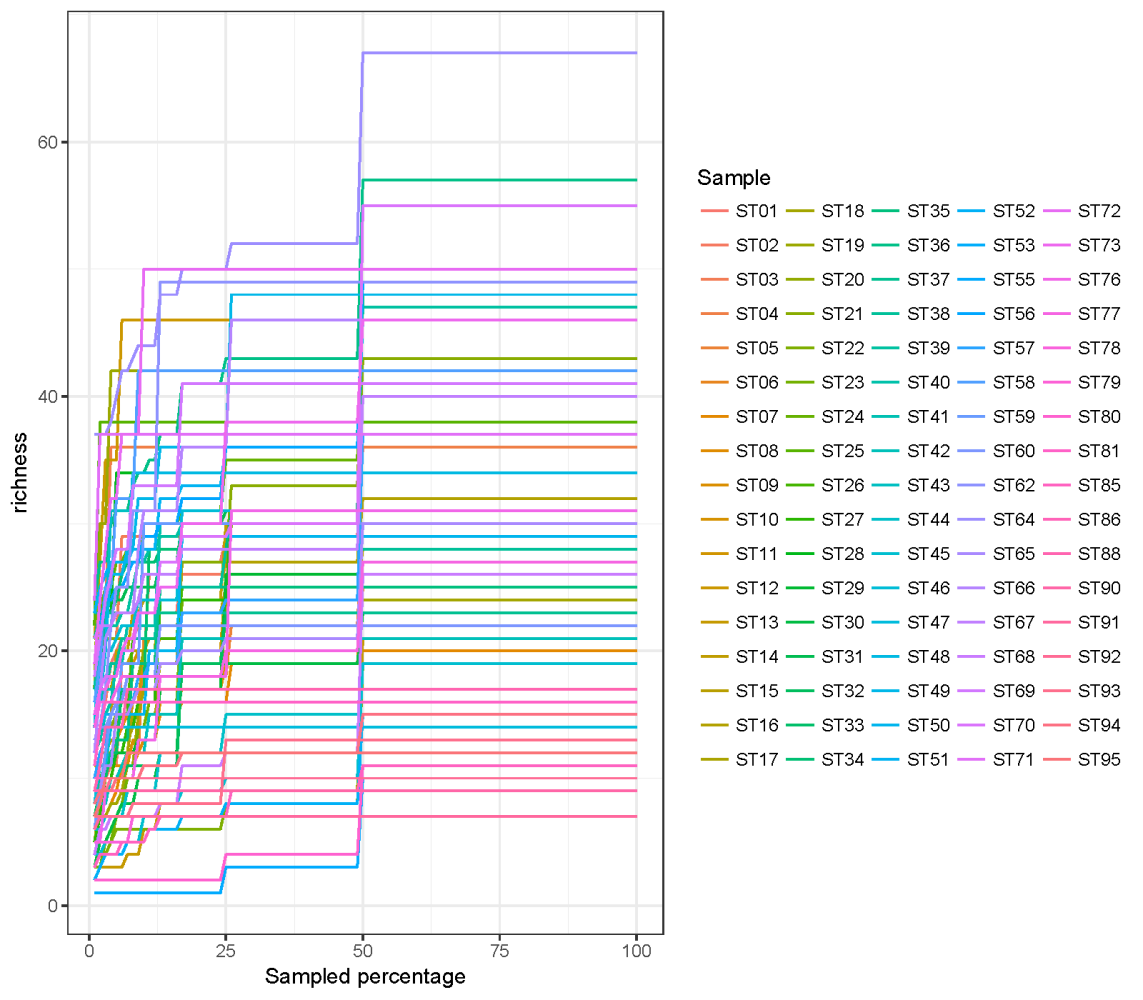 |
| --- |
| **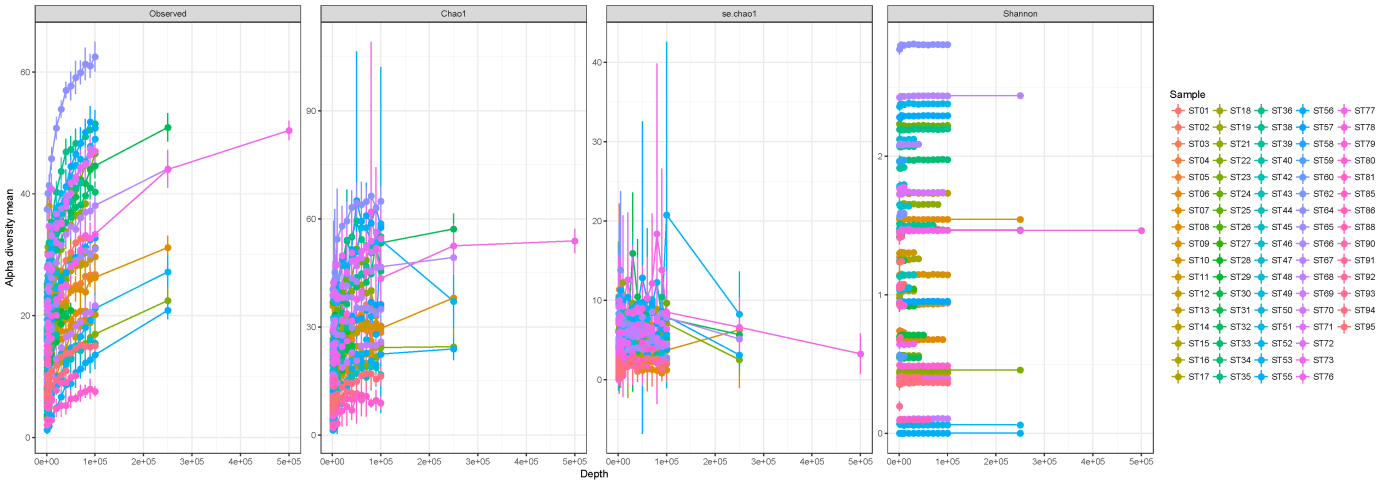** |
| **Figure S4** Rarefaction curves of the 86 sequenced samples. |

| 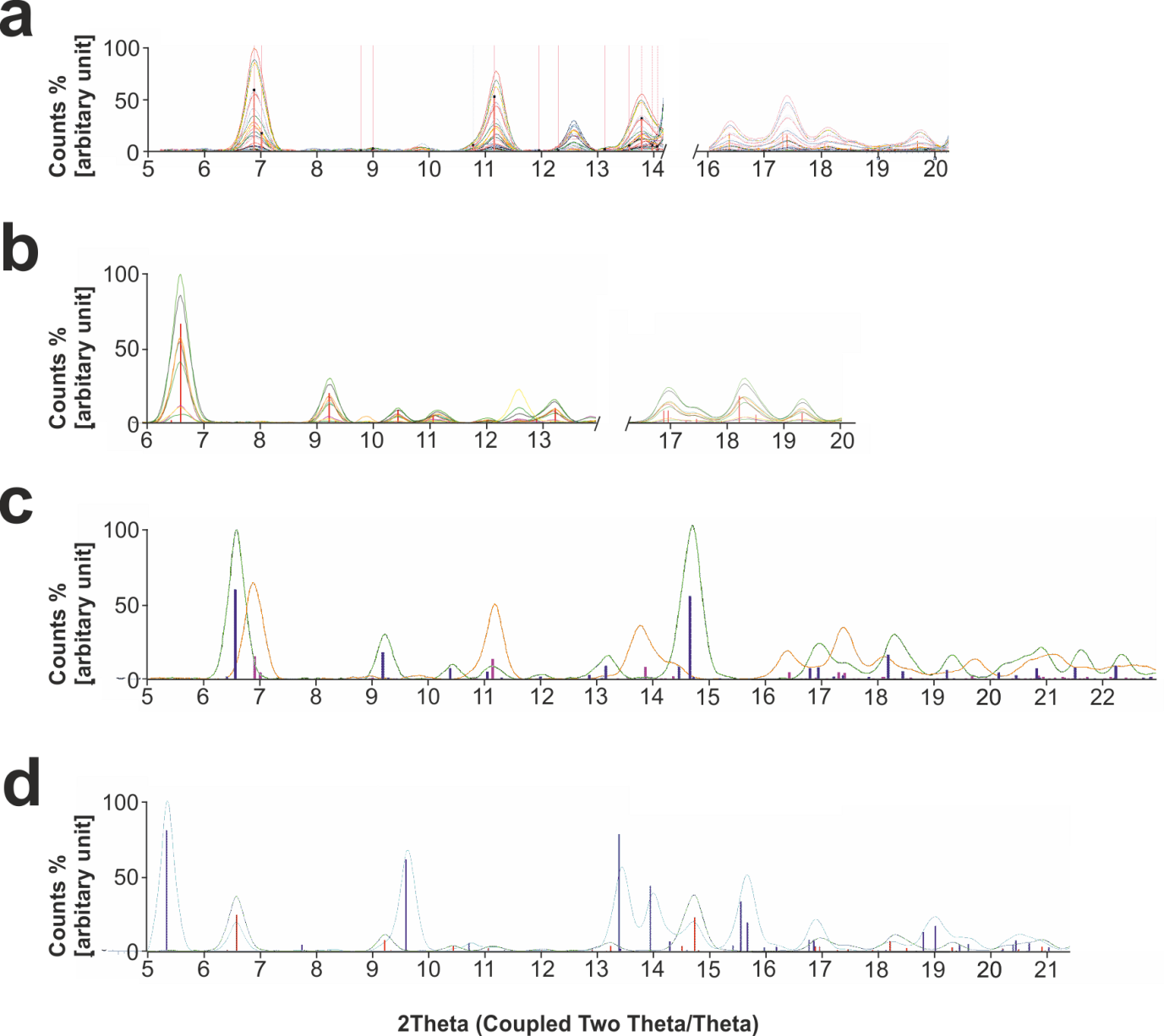 |
| --- |
| **Figure S5.** XRD signals and comparison with expected patterns from the COD database. **a** Overlay of XRD spectra of whewellite containing samples (λ = 0.71073Å). Red bars: expected whewellite diffraction pattern after applying “tune cell”. Cell parameters: Space group P 1 21/c 1 (14), a (Å): 6.26657, b (Å): 14.583, c (Å): 10.116; a/b: 0.42972; c/b 0.69368; β (°): 109.46. Signals at 2Theta of 12.5, 14.6 (cut out) derive from sodium chloride crystals formed during storage in saline solution. **b** Overlay of spectra of weddellite containing samples. Red bars: expected weddellite diffraction signals pattern after applying “tune cell”. Cell parameters: Space group I 4/m (87), a (Å): 12.32006, c (Å): 7.357; a/b: 1; c/b 0.59716. **c** Overlay of spectra of diffraction patterns a weddellite containing sample (green curve, ST12) with a whewellite containing sample (orange curve, ST19). Bars indicate characteristic diffraction patterns for weddellite (blue) and whewellite (margenta). **d** Overlay of the diffraction patterns of a dicalcium phosphate dehydrate and weddellite containing sample (blue curve, ST06) with a weddellite containing sample ST12 (green curve). All data are background subtracted. |
